# Post tracheostomy and post intubation tracheal stenosis: Report of 31 cases and review of the literature

**DOI:** 10.1186/1471-2466-8-18

**Published:** 2008-09-21

**Authors:** Nikolaos Zias, Alexandra Chroneou, Maher K Tabba, Anne V Gonzalez, Anthony W Gray, Carla R Lamb, David R Riker, John F Beamis

**Affiliations:** 1 Department of Pulmonary and Critical Care Medicine, Lahey Clinic Medical Center, Tufts University School of Medicine, Burlington, Massachusetts, USA; 2 Department of Pulmonary, Critical Care and Sleep Medicine, Tufts University School of Medicine, Boston, Massachusetts, USA

## Abstract

**Background:**

Severe post tracheostomy (PT) and post intubation (PI) tracheal stenosis is an uncommon clinical entity that often requires interventional bronchoscopy before surgery is considered. We present our experience with severe PI and PT stenosis in regards to patient characteristics, possible risk factors, and therapy.

**Methods:**

We conducted a retrospective chart review of 31 patients with PI and PT stenosis treated at Lahey Clinic over the past 8 years. Demographic characteristics, body mass index, co-morbidities, stenosis type and site, procedures performed and local treatments applied were recorded.

**Results:**

The most common profile of a patient with tracheal stenosis in our series was a female (75%), obese (66%) patient with a history of diabetes mellitus (35.4%), hypertension (51.6%), and cardiovascular disease (45.1%), who was a current smoker (38.7%). Eleven patients (PI group) had only oro-tracheal intubation (5.2 days of intubation) and developed web-like stenosis at the cuff site. Twenty patients (PT group) had undergone tracheostomy (54.5 days of intubation) and in 17 (85%) of them the stenosis appeared around the tracheal stoma. There was an average of 2.4 procedures performed per patient. Rigid bronchoscopy with Nd:YAG laser and dilatation (mechanical or balloon) were the preferred methods used. Only 1(3.2%) patient was sent to surgery for re-stenosis after multiple interventional bronchoscopy treatments.

**Conclusion:**

We have identified putative risk factors for the development of PI and PT stenosis. Differences in lesions characteristics and stenosis site were noted in our two patient groups. All patients underwent interventional bronchoscopy procedures as the first-line, and frequently the only treatment approach.

## Background

Post intubation (PI) tracheal stenosis was first recognized as an entity in 1880, after MacEwen instituted prolonged endotracheal intubation in four patients with upper airway obstruction[1]. Since then PI stenosis and later post tracheostomy (PT) stenosis has been rare but serious complications although the degree of tracheal stenosis may vary. Among all intubated patients, the reported incidence of PI and PT stenosis ranges from 10 to 22% [2-6] but only 1–2% of the patients are symptomatic or have severe stenosis [7-9]. Today, severe PI and PT stenosis are recognized entities with an estimated incidence of 4.9 cases per million per year in the general population [10].

Prolonged intubation can result in tracheal stenosis at various levels within the trachea[11]. Stenosis can occur anywhere from the level of the endotracheal tube tip up to the glottic and subglottic area, but the most common sites are where the endotracheal tube cuff has been in contact with the tracheal wall and at the tracheal stoma site after a tracheostomy procedure. Thus, tracheal stenosis can most commonly occur following the two types of airway intubation: endotracheal intubation (PI) and tracheostomy (PT).

Tracheal stenosis occurs at the endotracheal tube cuff site in one third of the reported PI cases[4,5,12] and appears as a web-like fibrous growth. The mainly postulated causative factor is loss of regional blood flow due to cuff pressure on the tracheal wall[13]. This ischemic injury begins within the first few hours of intubation, and healing of the damaged region can result in web-like fibrosis within 3 to 6 weeks[13,14]. Fortunately the advent of large volume, low pressure cuffs has markedly reduced the occurrence of cuff injury[6,15,16].

In contrast, tracheal stenosis following tracheostomy most commonly results from abnormal wound healing with excess granulation tissue formation around the tracheal stoma site; excess granulation tissue can also develop over a fractured cartilage, which can occur during the tracheostomy procedure[4,5,12,17]. Cartilage damage may also result from mechanical leverage of the tracheal tube at the stoma site due to the unsupported weight of ventilator attachments, causing pressure necrosis. In a recent review paper by Sarper et al. wound sepsis was also found as a causative factor in 42% of the stoma stenosis cases following open tracheostomy[18].

Multiple other factors predisposing to the development of PI and PT stenosis have been suggested, including: high tracheostomy site, prolonged intubation period, traumatic intubation, history of previous intubation or previous tracheostomy, excessive corticosteroid steroid usage, advanced age, female and estrogen effect, severe respiratory failure, severe reflux disease, autoimmune diseases (Wegener's granulomatosis, sarcoidosis and others), obstructive sleep apnea, and radiation therapy for oropharyngeal and laryngeal cancer[2,19].

In the past, tracheal stenosis was managed with dilatation alone[20,21], and success was measured by the ability to wean patients from their tracheostomy tubes. Until recently, surgical resection and end to end anastomosis was considered the only definitive treatment for tracheal stenosis[14,22]. Grillo and Mathisen[23] have reported low (1.8%) mortality rates associated with surgical intervention but others have reported mortality rates up to 5% [24,25]. Interventional bronchoscopy procedures can serve as a bridge to surgical treatment but most importantly, can constitute definitive therapy for many patients, including these that are surgical candidates[24]. Previous studies have reported variable success rates of interventional bronchoscopy procedures, ranging from 32[26] to 66%[27].

As an institution with a long experience in interventional bronchoscopy procedures, we felt it would be valuable to review our approach to the management of PI and PT stenosis. In this paper, we present the characteristics of patients with PI and PT stenosis treated at Lahey Clinic over the past 8 years, and review the treatment strategies used. Putative risk factors for the development of PI and PT stenosis are identified and discussed.

## Methods

Our institution serves as a regional referral center for interventional bronchoscopy procedures. We conducted a retrospective review of all patients who were referred to the interventional pulmonology service, department of pulmonary and critical care at Lahey clinic, Burlington, Massachusetts for evaluation and management of PI and PT stenosis. The study was approved by the Institutional Review Board.

Patients were identified from the prospectively maintained bronchoscopy suite and operation room logbooks. A total of 31 patients were identified between January 1999 and January 2007. Twenty patients were treated for tracheal stenosis following tracheostomy (PT group) and 11 patients were treated for tracheal stenosis developing after prolonged endotracheal intubation (PI group). Demographic data was obtained for each patient: age, sex, body mass index (BMI), co-morbid conditions and corticosteroid therapy received. The following data regarding the tracheal stenosis were also obtained: the circumstances leading to the development of tracheal stenosis (PI versus PT), type of stenosis (web-like stenosis, granulation tissue formation, tracheomalacia) and the therapeutic interventional pulmonary procedure(s) performed.

Each patient underwent a standard pre-operative assessment, including physical examination, routine laboratory tests, chest radiography and computed tomography of the chest. An initial diagnostic flexible bronchoscopy (FB) was performed for each patient to identify the type, location and severity of the stenosis. The stenosis was characterized severe if it was causing symptoms, primarily dyspnea, was complex in nature (stenosis combined with cartilage fracture or tracheomalacia) and the obstruction of the tracheal lumen exceeded 50%. The degree of stenosis was estimated with a dedicated instrument that was used to measure the diameter of the stenotic area and the diameter of the trachea lumen before and after the stenotic site. In some later cases the stenosis was estimated by virtual bronchoscopy along with the dedicated measuring device.

Rigid bronchoscopy (RB) was performed under general anesthesia in an operating room, while therapeutic FB was performed under moderate sedation with midazolam and fentanyl, in the bronchoscopy suite. The equipment used included the Dumon Series II rigid broncoscopes (Bryan Corporation Inc. Woburn, MA, USA) with optical system, stent introducer and forceps, and flexible bronchoscopes (Pentax Co. Tokyo, Japan or Olympus Co. Tokyo, Japan). Multiple therapeutic modalities were used including mechanical debulking and dilatation with the RB, balloon dilatation, Neodymium-Doped Yttrium Aluminium Garnet (Nd:YAG) laser photocoagulation, stent placement, electrocautery, and argon plasma coagulation (APC). During laser, APC and electrocautery treatment the FiO2 was adjusted to 30–40% to avoid the complication of endobronchial fire and burn.

For web-like stenoses a recommended mucosal sparring technique with radial incisions followed by airway dilatation was applied [27]. For stenosis produced by granulation tissue formation around the tracheal stoma, RB and photocoagulation with Nd:YAG laser was mostly used. As an alternative, electrocautery or APC was used under FB in some patients. Damaged cartilage and the remaining granulation tissue were removed by grasping forceps.

Stent implantation was used more commonly in patients with tracheomalacia and in patients with recurrent stenosis. Mixed stenosis was treated with more than one therapeutic modality.

Patients were considered cured when free of symptoms for at least one year after the initial intervention (the last treated patient was followed for 11 months). If re-stenosis was occurred on a follow up bronchoscopy (usually every 4 to 6 weeks for the first 6 months) then another intervention was applied. In most cases no more than 3 interventions were needed.

Numerical data are presented as means and standard deviation (or median and range) and were compared using the Student's t-test or the Mann-Whitney U-test when normality failed. Patient follow-up extended until November 2007. The statistics were computed with SPSS statistical software (SPSS version 14 for Windows; SPSS; Chicago, IL, USA).

## Results

PI and PT group characteristics are presented in Table 1. The patient population included 20 obese patients (some of them morbidly obese), raising the mean body mass index above 30 in both groups. Patients were predominantly female in both groups. The most frequent co-morbidities were diabetes mellitus, cardiovascular disease, hypertension and gastro-esophageal reflux disease. One third of the patients were current smokers.

**Table 1:** Patients characteristics and co-morbidities as seen in both patient groups at the time of the first interventional bronchoscopy procedure performed.

	PT group (n = 20)	PI group (n = 11)
Male/female*	7/13	1/10
Age (median-range)	58.2 (28–86)	50.2 (21–76)
Body Mass Index (mean ± SD)	34.1 ± 8.9	32.3 ± 9.6
Diabetes mellitus	8 (40%)	3 (27%)
Cardiovascular disease	9 (45%)	5 (45%)
Coronary Artery Bypass Graft	3 (15%)	2 (18%)
Systemic Hypertension	11 (55%)	5 (45%)
Gastro Esophageal Reflux Disease	6 (30%)	2 (18%)
Chronic Obstructive Pulmonary Disease	3 (15%)	2 (18%)
Asthma	2 (10%)	2 (18%)
Obstructive sleep apnea	3 (15%)	0
Hypothyroidsm	3 (15%)	0
Alcohol	4 (20%)	2 (18%)
Smoking	7 (35%)	5 (45%)
Corticosteroid therapy	2 (10%)	2 (18%)

*female predominance is seen in both groups, PI: post intubation, PT: post tracheostomy, SD: standard deviation.

The characteristics of the tracheal stenosis varied depending on whether the development of tracheal stenosis followed post intubation or tracheostomy, and are presented in table 2. The mean length of the stenosis was greater in the PI group due to the (common) formation of a web-like stenosis along the distribution of the endotracheal tube cuff. Patients with PI stenosis had a mean duration of intubation of 5.2 days. Not surprisingly, patients with tracheal stenosis following tracheostomy had a much longer duration of cannulation, with a mean of 54.5 days.

**Table 2:** Characteristics of the tracheal stenosis regarding days of intubation, the exact site and degree of the stenosis.

	PT group (20 patients) (mean-range)	PI group (11 patients) (mean-range)
Days with ETT	5.5 (4–7)	5.2 (2–10)
Days with tracheostomy	54.5 (14–151)	-
Distance from vocal cords (cm)	3.1 (2–7)	2.7 (1–5)
Distance from main carina (cm)	5.5 (5–6)	5.5 (3–8)
Length of stenosis (cm)	1.2 (0.5–2.5)	2.6 (0.5–7)
Percent of tracheal stenosis	78.5% (60–90%)	71% (50%–90%)

ETT: endotracheal tube, pts: patients, PI: post intubation, PT: post tracheostomy.

**Figure 1:**
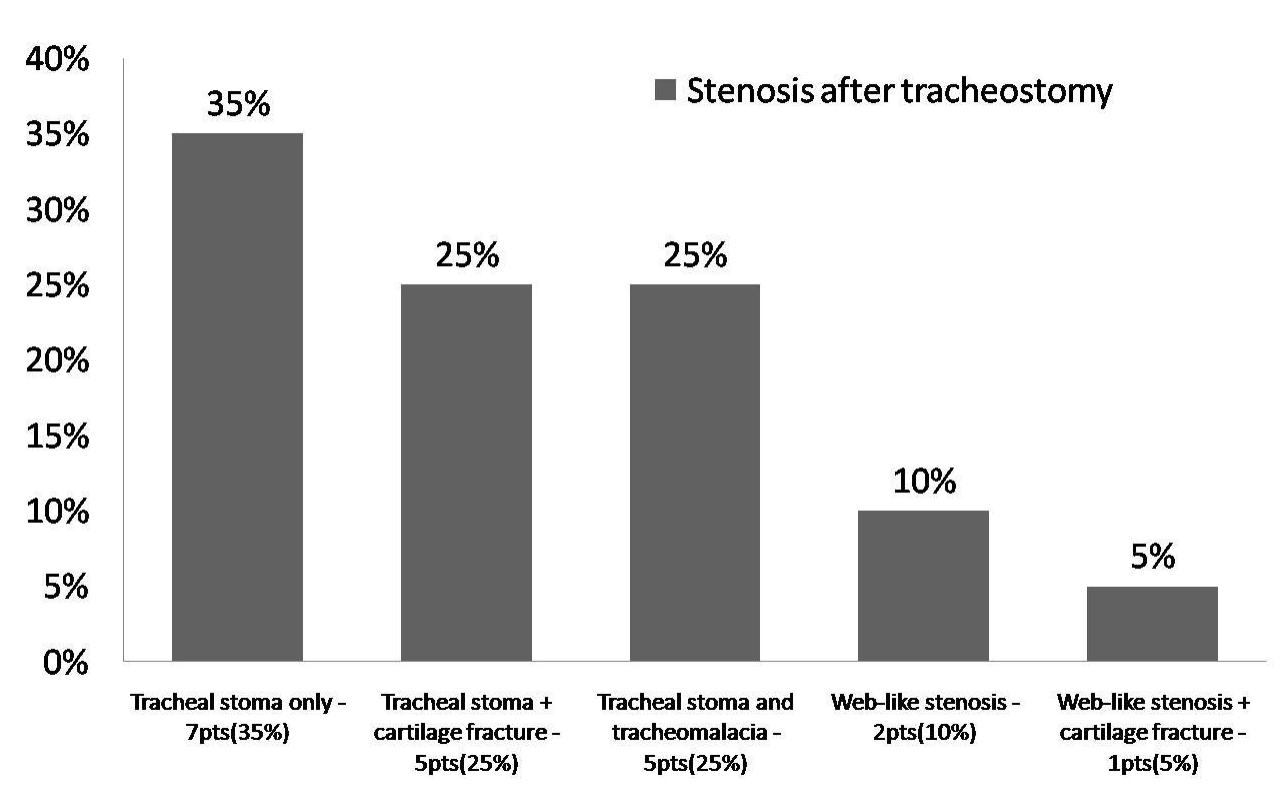
Site and type of the stenosis in the post tracheostomy group.

**Figure 2:**
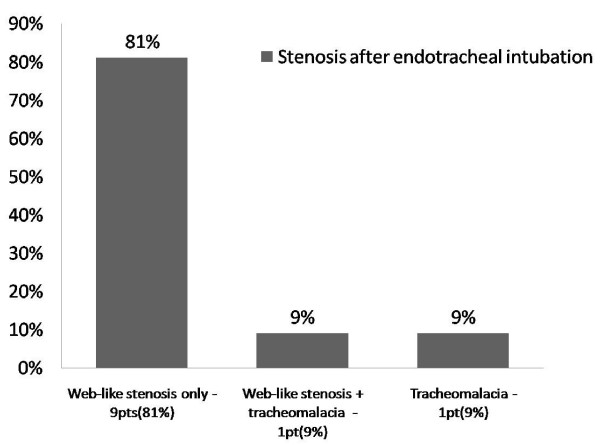
Site and type of the stenosis in the post intubation group.

The types of the tracheal stenoses developing in the PT and PI groups are presented in Figures 1 and 2. The most common type of stenosis was granulation tissue formation at the stoma site in the PT group, and web-like fibrosis in the PI group. No significant difference was noted between males and females with regards to the predisposing factors or co-morbid conditions, and the type of tracheal stenosis (PI ot PT).

The treatment modalities used are presented in Table 3. RB was the most commonly utilized procedure to approach the airway with tracheal stenosis. Mechanical debulking and dilatation with the RB, balloon dilatation, Nd:YAG laser therapy and stent placement were the most common interventional procedures employed.

**Table 3:** A variety of modalities and treatments applied in both groups with the use of rigid as well as the flexible bronchoscopes.

	PT group (n = 20)	Number of procedures	PI group (n = 11)	Number of procedures
RB and FB (mean and range)	20	41 (2.1, 1–5)	11	36 (3.2, 1–17)
Surgery	0	0	1	1
Nd:YAG Laser	8	9	6	14
Electrocautery	5	7	0	0
APC	1	1	2	2
Balloon dilatation	6	8	3	3
Cryotherapy	1	1	1	1
Stent	11	15	6	11
Montgomery tube	2	2	0	0
Mechanical debulking/dilation	5	5	6	7

APC: Argon Plasma Coagulation, Nd:YAG: Neodymium-Doped Yttrium Aluminium Garnet, PI: Post Intubation, PT: Post Tracheostomy. RB: Rigid bronchoscopy, FB: Flexible bronchoscopy.

Each patient underwent an average of 2 procedures during their treatment course. Most of the patients underwent 1 to 4 procedures; two patients were treated with 5 procedures and a single patient who was eventually sent to surgery underwent a total of 17 procedures. This patient was sent to surgery because of recurrent tracheal stenosis after multiple treatments with rigid bronchoscopy, Nd:YAG laser and repeated silicone stent implantations over a six-year period. This patient was a female, obese patient (BMI: 33.4) who developed tracheal stenosis after 4 days of endotracheal intubation for MRSA pneumonia. She had multiple risk factors including sickle cell anemia, diabetes mellitus and asthma and was receiving 30 mg po daily of prednisone.

Eight patients (26%) developed recurrent tracheal stenosis after the first bronchoscopic intervention, with 7 of them being females. No correlation was found between the tendency to develop re-stenosis and the type of the stenosis (PI or PT), the potential risk factors identified, or the treatment modality used.

## Discussion

In this study, we have reviewed the patient characteristics and endoscopic treatments performed in a series of individuals who developed tracheal stenosis following endotracheal intubation or tracheostomy. Our study results show the similarities of characteristics among the two patient groups and point out the differences in the stenosis type/site and treatments applied between them. An important limitation is the lack of a control group that would have allows us to estimate more accurate the risk factors and patients characteristics that lead to tracheal stenosis. The reason for difficulty selecting an appropriate control group is the fact that our patients were referred to us from different hospitals or even from different states, so we were not able to find a control group of patients that would resemble our patients' characteristics.

Our paper differs from others published in the literature regarding patients' profile. The typical profile of a patient with PI or PT stenosis that emerged from our data is a female obese patient with past medical history of diabetes mellitus, hypertension, and cardiovascular disease, who was a current smoker. The gender influence has been controversial in the literature. A predominance of female with tracheal stenosis has been reported in two series by McCaffrey et al[28] and Mehta et al[27] respectively. Female predominance is also reported in cases of idiopathic subglottic stenosis[19,29,30]. It has been postulated that estrogens increase the levels of transforming growth factor β1 promoting extracellular matrix production, including the deposition of type I and III collagens and finally fibrosis. Whether this mechanism could contribute to the female predominance noted in our cases of tracheal stenosis is unclear.

All of our patients had severe (>50%) tracheal stenosis with an average degree of stenosis of more than 70%. Severe tracheal stenosis prevalence should be very low [7-9] especially since the introduction of large volume, low pressure endotracheal tube cuffs, the careful placement of the tracheostomy stoma, avoidance of large apertures, elimination of heavy ventilatory connecting equipment, and meticulous care of the tracheostomy[6,15,16]. A study by Norwood et al[31] who followed 48 patients for 30 months after percutaneous tracheostomy using tracheal CT scans found that only 1 patient (2%) developed severe tracheal stenosis, while mild to moderate stenosis was detected in 14 (29.3%) patients. Our series reflects a large referral network and does not necessarily reflect the true prevalence of the condition.

The site of the stenosis varies according to whether the patient has had tracheostomy or only endotracheal intubation. Patients with PI stenosis tend to develop web-like fibrous stenosis at the cuff site while tracheostomy patients develop stenosis due to granulation tissue around the stoma site, and frequently there is an associated cartilage fracture or malacia of the trachea wall. Also patients in the tracheostomy group were intubated for longer periods, thus exposing them to more trauma at the tracheal stoma site, and risk of infection. The mean length of the web-like tracheal stenosis lesion (2.6 cm) in the PI group is in accordance with the results of other case series[28,32,33]. The length of stenosis in the PT group was only 1.2 cm. Stenosis that developed as a web around an endotracheal tube cuff is longer and more uniform than the stenosis around a tracheal stoma where granulation tissue can extent from a fissure in the anterior trachea or grow into a bulky granulomatus formation surrounding a fracture cartilage.

Obesity was mentioned as a coexisting underlying condition in 14% of the cases described by Cavaliere at al[34]. No studies in the literature have previously correlated obesity with the risk of developing tracheal stenosis. The rate of obesity (66%) in our case series exceeds the National Health and Nutrition Examination Survey prevalence of obesity in adults, which was estimated at 32% in 2004 [35-37]. Obesity correlates also with an increased neck circumference that poses a higher risk of trauma and cartilage fracture during a tracheostomy procedure.

Diabetes mellitus prevalence in patients with PI or PT stensois ranges from 10 to 23% in several case series[19,24,34] while cardiovascular disease ranges from 17.5 to 46%[24,32,34]. In our patient groups diabetes mellitus and cardiovascular disease appear as co-morbidities in at least one out of three patients. Patients with diabetes mellitus and/or cardiovascular disease may have microvascular occlusion that contributes to the regional ischemia caused by the endotracheal tube cuff pressure[14]. The same effect of regional ischemia might be expected during situations of low perfusion pressure such as cardiopulmonary bypass[14]. In our study 5 patients (16%) developed tracheal stenosis after cardiopulmonary bypass surgery.

Smoking history have been suggested as potential risk factor by Koshkareva et al[19] but other studies have shown no significant correlation with the risk of developing tracheal stenosis[18,33,34].

In our patients corticosteroids equivalent to 10–50 mg prednisone treatment for co-morbidities such as idiopathic pulmonary fibrosis (IPF), cryptogenic organizing pneumonia (COP) or sickle cell anemia was recorded in 4 (12.9%) patients. Steroids, because of their effect on wound healing, have been reported as a predisposing factor for the development of tracheal stenosis. This observation, however, has not been universally accepted[2].

Seven (22.5%) of our patients had a history of reflux disease, which has previously been reported only as a risk factor for idiopathic subglottic stenosis[38,39].

The management of patients with PI or PT stenosis varies according to the location of the damage, the severity of the stenosis, the initial airway injury trigger (endotracheal tube vs tracheostomy), the subsequent stenosis type and the presence of co-morbid conditions. Our treatment approach was different between the two groups. Web-like stenosis in PI patients was treated with radial incisions (laser, electrocautery or APC) and dilatation. Cartilage fracture in the anterior tracheal wall after a tracheostomy procedure was most likely to happen in patients with calcified cartilage rings and was visible as a white cartilaginous material protruding into the tracheal lumen. Granulation tissue and/or damaged cartilage in PT patients was mainly photocoagulated by Nd:YAG laser during a rigid bronchoscopy procedure and removed with the use of biopsy forceps. In some cases, APC and electrocautery modalities were used under flexible bronchoscopy procedure and this is also the approach followed and described in several other articles for granulation tissue removal[24,40]. Stent implantation was used as a last therapeutic resort in both patient groups. Several rigid bronchoscopy and/or flexible bronchoscopy procedures were often required to achieve optimal results. One patient underwent 17 rigid bronchoscopies and was ultimately sent to surgery. Our general approach is to manage PI and PT stenosis non-surgically, and reserve the surgical option for recurrent and refractory cases. Surgery is not always feasible, because of co-morbidities and poor performance status[24]. Brichet et al[24] described their multidisciplinary approach for post intubation tracheal stenosis, and reported that surgery was required in only 2 patients (8%) with web stenosis and 2 patients (13%) with complex stenosis.

Re-stenosis at the site of the intervention (as a result of an abnormal healing process) or a stent "event" (obstruction, migration or halitosis) were the most common reasons of a multiple procedure. The mean number of treatments provided to our patients (2.1 in PT group – 3.2 in PI group) is higher than that reported by others (1.5–1.7 treatments) [19,27]. (The fact that our patient underwent 17 procedures has skewed our results. If this patient were excluded from the analysis the mean number of procedures performed would have dropped to 1.9 for the PI group.)

In regard to therapy most prior reports on this subject combine these two patient groups because treatment is similar and relies on interventional bronchoscopy procedures or surgery. We postulate that the etiology, pathogenesis and treatment approach of the stenoses differ significantly between the PT and PI group.

In comparison with other series our study highlights the differences in patient characteristics and treatments approach between the two types of stenoses. Due to the rarity of tracheal stenosis, all case series report a small number of patients often with characteristics that differ between institutes. Although there is no uniform treatment approach to these patients, most centers tend to rely on surgery for the treatment of the stenosis. The interventional bronchoscopy is relatively new and now evolving. Although surgery is a definitive treatment, patients with co-morbidities and poor performance status may not be eligible for this option. Mortality rates after end to end anastomosis can be seen up to 5% [24,25] along with complications such as re-stenosis, suture granuloma formation, infections, hemorrhage, subcutaneous emphysema and others[41,42]. As we have already mentioned co-morbidities are almost always present in PI and PT patients and thus interventional bronchoscopy procedures may be the only available option.

## Conclusion

Tracheal stenosis after endotracheal intubation and tracheal stenosis after tracheostomy differ in etiology and pathogenesis and should be considered as two different entities.

Interventional bronchoscopy should be the first approach in the treatment sequence of these patients, and may be the only required treatment in the majority of patients.

Prospective carefully designed controlled studies are needed to better define the role of predisposing factors and co-morbidities in determining appropriate treatment for tracheal stenosis.

## Abbreviations

APC: Argon Plasma Coagulation; BMI:Body Mass Index; COP: Cryptogenic Organizing Pneumonial; CT:Computed Tomography; FB: Flexible Bronchoscopy, FiO2: Fraction of Inspired Oxygen; IPF:Idiopathic Pulmonary Fibrosis; MRSA: Methicillin-Resistant Staphylococcus Aureus; Nd:YAG: Neodymium-Doped Yttrium Aluminium Garnet; PI: Post Intubation; PT: Post Tracheostomy; RB: Rigid Bronchoscopy.

## Competing interests

The authors declare that they have no competing interests.

## Authors' contributions

JFB, AWG, CRL, and DRR performed the procedures during this 8 year period. JFB, AVG and MKT participated in the design of the study and reviewed the manuscript thoroughly. NZ and AC reviewed the charts, created the database, performed the statistical analysis and wrote the manuscript.

## Pre-publication history

The pre-publication history for this paper can be accessed here:


